# Sensitization or inoculation: Investigating the effects of early adversity on personality traits and stress experiences in adulthood

**DOI:** 10.1371/journal.pone.0248822

**Published:** 2021-04-01

**Authors:** Jing Luo, Bo Zhang, Brent W. Roberts

**Affiliations:** 1 Department of Medical Social Sciences, Feinberg School of Medicine, Northwestern University, Chicago, IL, United States of America; 2 Department of Brain and Psychological Sciences, Texas A & M University, College Station, TX, United States of America; 3 Department of Psychology, University of Illinois at Urbana-Champaign, Champaign, IL, United States of America; University of Sao Paulo Medical School, BRAZIL

## Abstract

Cumulative evidence has been found for the associations between personality traits and stress experiences in adulthood. However, less is known about the moderating mechanisms underlying these associations. The present study tested whether the stress sensitization and stress inoculation hypotheses could be applied to the relationship between early adversity and personality in adulthood. Specifically, we tested the linear and curvilinear relations between early adversity (measured retrospectively) and adulthood personality traits, as well as the linear and curvilinear moderating effects of early adversity on the associations between adulthood stress and personality traits. Samples of older adults from the Health and Retirement Study (HRS; N = 6098) and middle-aged adults from the Midlife in the United States Survey (MIDUS; N = 6186) were used. Across the two samples, positive linear associations were found between retrospective early adversity and neuroticism. The results also suggested significant linear effects of early adversity on the association between ongoing chronic stressors and neuroticism such that individuals with moderate exposure to early adversity showed stronger associations between ongoing chronic stressors and neuroticism. Results from the current research were more in line with the stress sensitization model. No support was found for the stress inoculation effects on personality.

## Introduction

The preponderance of research suggests that stress has crucial impact on various life outcomes [1–4]. In search of individual characteristics that relate to stress experiences, personality traits have been shown to be associated with both exposure to stressors and subjective perceptions of stress [5, 6]. However, developmental factors that may influence the associations between personality traits and stress experiences during adulthood remain largely unknown.

Accumulated evidence has suggested early adversity to be one of the most prominent developmental factors that predisposes individuals to heightened risk for a variety of negative outcomes later in life [7–9]. According to previous research, early adversity has been defined as experiences that may acquire significant adaptation by an average child and that represent a deviation from the expectable environment [10]. Theoretical models have been devoted to understanding the process of how early adversity may impact different outcomes in adulthood, two of which are the stress sensitization and the stress inoculation models. The stress sensitization model suggests that the experiences of adversity early in life lead to heightened reactivity to subsequent stressful events, increasing the likelihood of negative consequences [11, 12]. In contrast, according to the stress inoculation hypothesis, a curvilinear relationship between early adversity and adulthood outcomes may be present such that exposure to moderate levels of adversity early in life may promote the development of resilience and protect individuals from potential negative consequences, while extremely low and high levels of exposure to early adversity may make individuals more vulnerable [13–15].

The current study aimed to test the two competing theoretical models for how the experiences of early adversity may be associated with the personality traits, and the associations between stress experiences (including both exposure to stressors and perception of stress) and personality traits in adulthood.

### Stress sensitization vs. stress inoculation

Originally presented as a theory to explain the role of early adversity in the vulnerability to depression, the stress sensitization model proposes that the adverse experiences sensitize individuals to heightened sensitivity and reactivity to subsequent stress [11, 12], resulting in an increased risk for depressive symptoms. In accordance with the stress sensitization hypothesis, adversity in early life was also found to increase the risk of other mental disorders (e.g., post-traumatic stress disorder (PTSD), anxiety disorders, bipolar disorder, and disordered drug use [12, 16, 17]) in response to stress experiences later in life. Furthermore, for those who suffered from higher levels of early adversity [18, 19], the experiences of stressor in adulthood were more strongly associated with elevated risk of intimate partner violence, as well as lower likelihood of smoking cessation when compared with participants with lower levels of early adversity.

In contrast to the stress sensitization model, the stress inoculation model assumes a more complicated relation between early stress and outcomes later in life. Rather than positing a monotonic relationship between early adversity and reactivity to stress later in life, the stress inoculation model assumes a curvilinear relation between stress exposure early in life and later reactions to stress such that exposure to a moderate level of early adversity may confer protective effects, rather than vulnerability, to subsequent stress and its potential detrimental consequences [13–15]. Specifically, according the stress inoculation hypothesis, a U-shape relationship is expected between early adversity and subsequent negative outcomes (e.g., neuroticism, health problems), while an inverted U-shaped association is suggested for early adversity and subsequent positive outcomes (e.g., conscientiousness, well-being). As suggested by the stress inoculation model, relatively moderate stress (compared to low or severe levels) is not overwhelming but is sufficiently challenging for the development of emotional and physiological resources to better cope with future stress experiences. Thus, a moderate level of early adversity has some “tempering” effects that protect individuals from future stress and its potential negative influences [20, 21]. Low levels of early adversity are not sufficiently arousing to stimulate the development of relevant resources, leaving the individual unprepared and sensitive to future stressful experiences. In contrast, severe adversity in early life overwhelms and makes the individual unable to manage future stressful situations [22, 23]. Evidence supporting the stress inoculation model has been reported in previous studies. For example, individuals with a history of some early adversity or lifetime adversity were found to report better mental health and well-being than individuals experienced a high level of adversity or those with no history lifetime adversity [24–26]. Other studies reported quadratic relationships between early life stress and depressive response to proximal negative events and implicit anxiety [24, 26]. Evidence has also been found for curvilinear relations between life stress and health outcomes in adulthood. For example, in a sample of subjects suffering from chronic back pain, significant U-shaped quadratic relationships emerged between histories of lifetime adversity and different health outcomes [27].

Some studies tested both the stress sensitization and the stress inoculation hypotheses. For example, a study examined the impact of early parental loss on emotional reactions and physiological reactions to subsequent minor stress in late adolescence/young adulthood reported support for both the stress sensitization and the stress inoculation models [28]. Specifically, it was suggested that the stress inoculation model was supported by the findings that individuals who experienced parental bereavement displayed lower blood pressure than those in the nonbereaved group. However, within the bereaved group, results indicated that individuals with lower perceived caring from the surviving parent showed higher levels of negative emotional reactions to stress than those without. Given that within the bereaved group, individuals perceived lower parental caring experienced higher levels of early adversity compared to others in this group, the results were interpreted as evidence for the stress sensitization hypotheses (higher early adversity was related to more negative emotional outcomes). In a study that examined the role of childhood social stress in depressive reactions to subsequent interpersonal stress in two independent samples, evidence was found in pubertal girls and prepubertal boys for the stress sensitization processes but not the stress inoculation process [29]. However, in both studies, the relationships between adversity in childhood and stress later in life and reactions to the later stress were only tested in linear but not curvilinear models. As the stress sensitization and the stress inoculation models suggest differential patterns of effects (linear vs. curvilinear) that early adversity may have on subsequent outcomes, it is important to test the effects of early adversity using both the linear and curvilinear models.

Thus, previous research has found some support for both stress sensitization and stress inoculation models. What is unknown presently is whether the ideas behind the stress sensitization and stress inoculation hypotheses can also be applied to individual differences in personality at middle and late ages as well as the relations between personality traits and stress at middle and late ages.

### Early adversity and adulthood personality

Personality traits are defined as the relatively enduring, automatic patterns of thoughts, feelings, and behaviors that distinguish individuals from one another and that are elicited in relevant situations [30]. Among the models describing the structure of personality traits, the Big Five personality model has become the most widely accepted framework [31], with the five personality domains commonly labeled as neuroticism, extraversion, agreeableness, conscientiousness, and openness to experience.

Previous studies have examined the associations between early adversity and personality traits in adulthood. Individuals who experienced childhood adversity have been found to display significantly higher levels of neuroticism and openness, but lower levels of conscientiousness (M_age_ = 32) [32]. Similar findings were suggested by a second study in which subjects exposed to high levels of early life stress were found to endorse higher neuroticism and openness; however, other dimensions of the Big Five were not found to be affected by early life stress (M_age_ = 39) [33]. The third study that examined the link between early adversity and personality traits reported early adversity to be related to greater levels of anger and aggression, lower levels of agreeableness, and higher levels of extrinsic focus (M_age_ = 19) [34].

Thus, according to previous studies, early adversity has been shown to be associated with less adaptive personality traits, which is consistent with the stress sensitization hypothesis. However, it remains unclear that whether early adversity was still related to personality traits when exposure to stress in adulthood is taken into consideration as early adversity and adulthood stress exposure have been found to be correlated [35]. Furthermore, all of the reported associations were examined under the assumption that linear relations exist between early adverse experiences and personality traits later in life. No study has tested the possible curvilinear associations between early adversity and personality traits in adulthood by examining the effects of the quadratic form of early adversity (under the assumption of the stress inoculation model). Given the hypothesized inoculating effects of early adversity, it is possible that exposure to moderate levels of adversity early in life benefits the development of personality traits in adulthood and protects personality development from the influences of subsequent stressful life events. Also, generally, previous studies tested the relationships between early adversity and adulthood personality traits in relatively young samples. It is still unclear whether the associations between early adversity and personality traits can be replicated in middle-aged and older adults.

### Stressor exposure, perceived stress, and personality traits in adulthood

Stressor exposure, the external environmental threats or challenges to which individuals are exposed [36], has been shown to be related to personality traits development over time. It has been suggested that exposure to stressful events impacts personality traits in a bottom-up fashion such that individuals may exhibit prolonged changes in emotions, thoughts, and behaviors in response to stressful experiences [37]. A study reported that individuals who experienced extremely horrifying or frightening events displayed increased neuroticism, decreased compliance (facet of agreeableness), and decreased openness relative to those without the experiences [38]. Increased conscientiousness was found in women as a response of widowhood, whereas men displayed decreased conscientiousness after the death of a spouse [39]. Other studies reported the relationships between the experience of unemployment and personality traits. For example, it was found that individuals who were fired displayed increases in neuroticism and decreases in conscientiousness when compared to those who were promoted [40]. Results from a study that suggested significant associations between unemployment and personality traits, with the influences of unemployment contingent upon gender, the number of years of unemployment, and the experiences of reemployment [41]. Therefore, according to previous research, personality traits is related to exposure to stressful experiences, and the relationship between stressor exposure and personality traits may vary on the basis of other factors (such as gender and widowhood reviewed above).

In addition to exposure to stressors, perception of stress, individuals’ psychological reactions to stressors, was also found to be related to personality traits. Personality traits have been suggested to be strongly related to both the descriptive situational representations of stress and the evaluative aspects of the perceptions of stressful situations [42]. Specifically, neuroticism is related to negative descriptions of the environment, while conscientiousness and agreeableness were found to be associated with positive perceptions of the environment. For subjective evaluations, individuals high on neuroticism were more likely to interpret everyday situations as threatening or damaging, and they were prone to appraise their susceptibility to health risks as higher. In contrast, individuals high on conscientiousness, extraversion, and agreeableness view mundane situations as less threatening and perceive their vulnerability to health risks as lower. Consistently, neuroticism was found to be positively related to perceived stress, while extraversion, agreeableness, conscientiousness, and openness displayed negative correlations with perceived stress [43–45].

Therefore, according to previous research, personality traits and stress experiences in adulthood are associated with each other. However, much less is known about whether the association between personality traits and stress experiences may vary on the basis of other factors. Specifically, it remains unclear whether early life adversity is related to the stress-personality relation in adulthood.

### The current study

Evidence has been found for both the stress sensitization and stress inoculation hypotheses for the effects of early adversity on important life outcomes in response to subsequent stress experiences. However, less is known about how early adversity may be associated with personality traits and personality traits in response to the experience of stress. The current research is the first study investigated the linear and curvilinear relations between early adversity and personality traits in adulthood after controlling for adulthood stressor exposure, as well as the linear and curvilinear moderating effects of early adversity on the associations between adulthood stress experiences and personality traits in two large representative samples. As the effects of early adversity in prior research have been mainly tested in adolescent and young adult samples, the present study examined whether early adversity still displayed relationships to personality traits and the associations between stress and personality traits in middle-aged and older samples.

**Fig 1:**
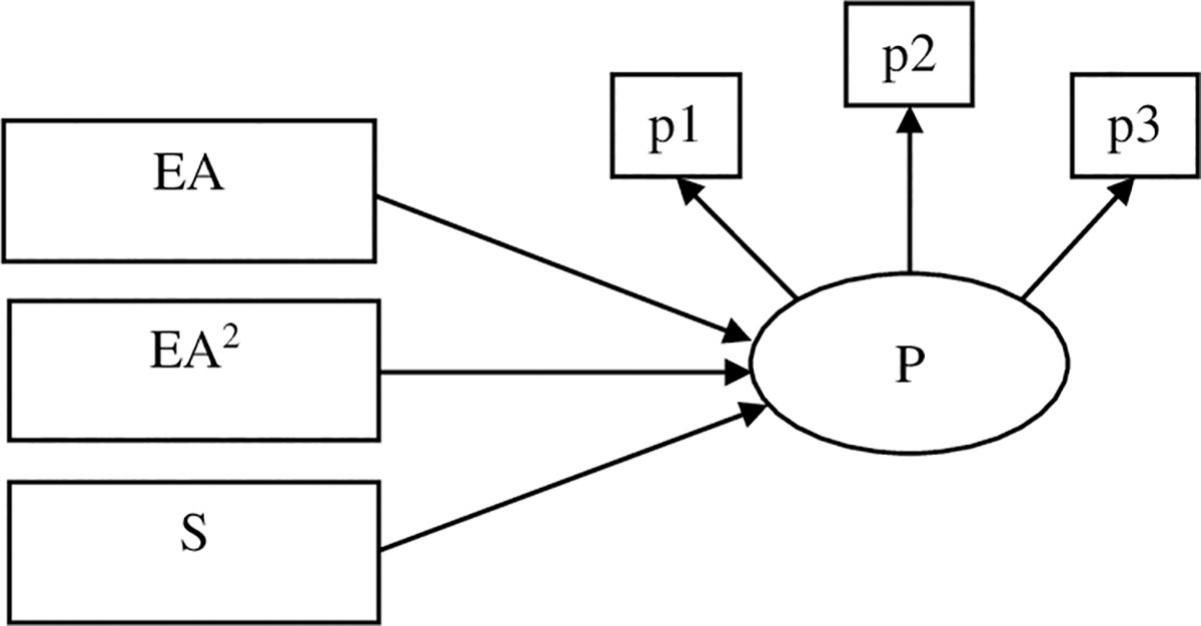
The conceptual diagram for the test of linear and quadratic effects of early adversity on personality traits. P = personality traits; EA = early adversity; S = adulthood stressor exposure; p1-p3 represents manifest indicators of personality.

**Fig 2:**
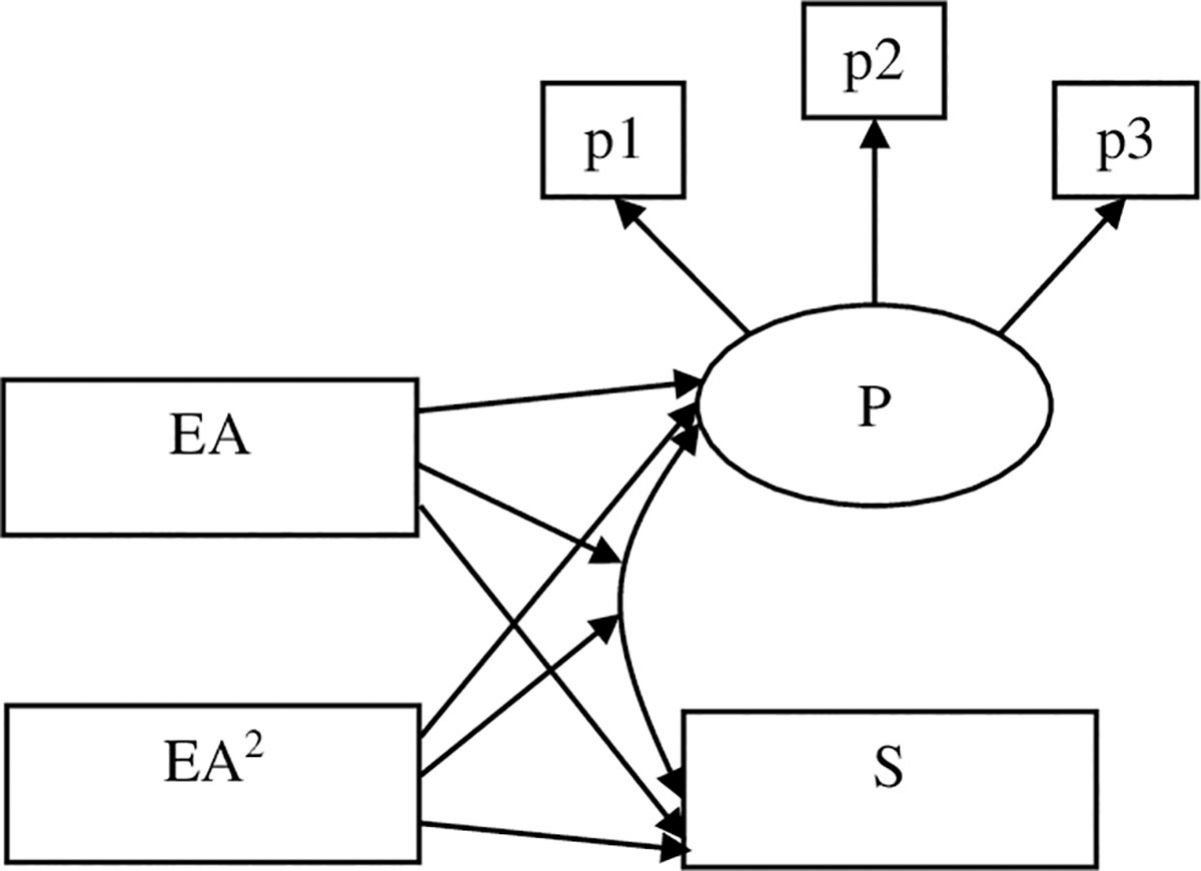
The conceptual diagram for the test of the linear and quadratic moderating effects of early adversity on the covariance between adulthood stressor exposure and personality traits. P = personality traits; S = stressor exposure; EA = early adversity; p1-p3 represents manifest indicators of personality.

**Fig 3:**
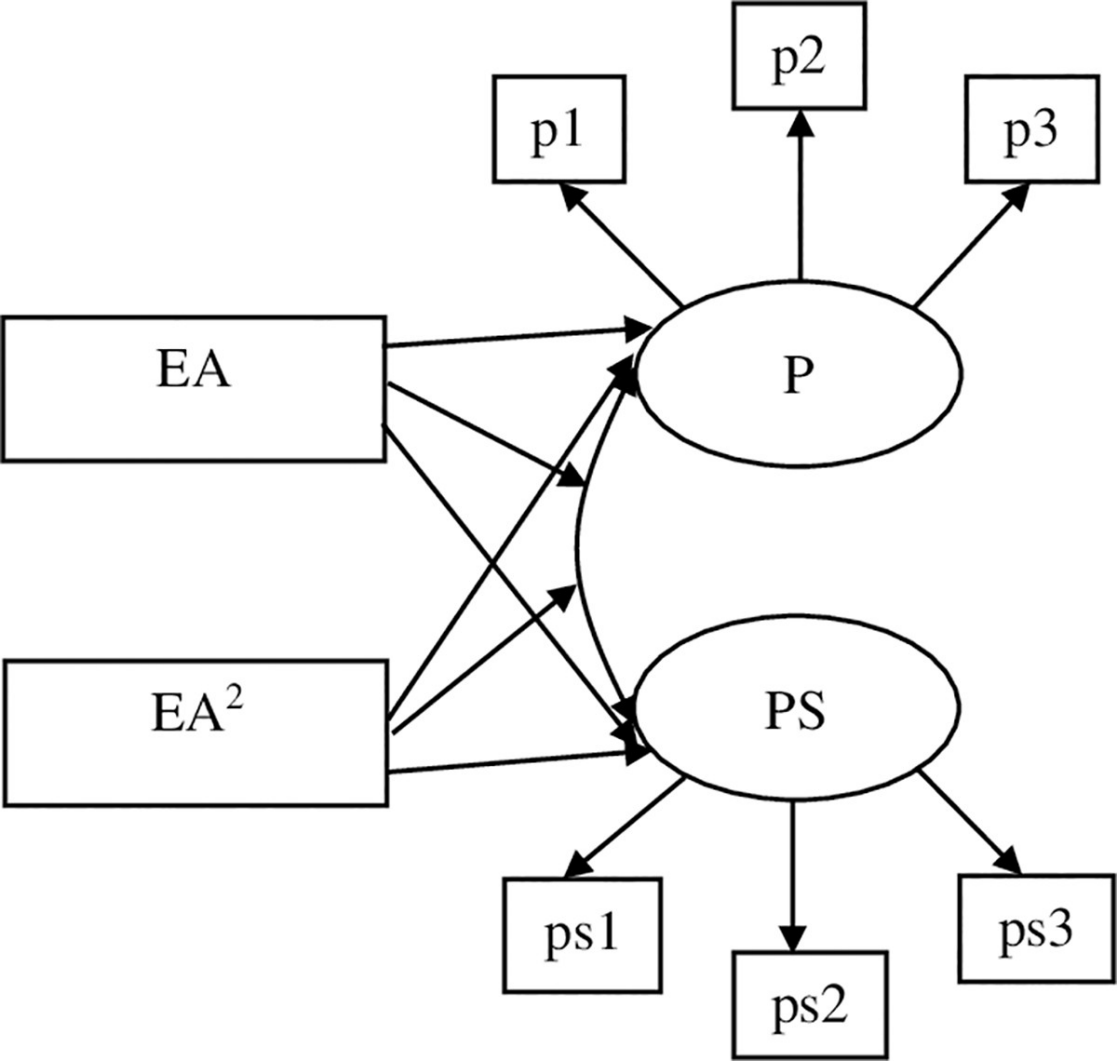
The conceptual diagram for the test of the linear and quadratic moderating effects of early adversity on the covariance between adulthood perceived stress and personality traits. P = personality traits; PS = adulthood perceived stress; EA = early adversity; p1-p3 represents manifest indicators of personality; ps1-ps3 represents manifest indicators of perceived stress.

Specifically, the current study first aimed to test the linear and curvilinear relations between early adversity and the Big Five personality traits in mid and late adulthood after controlling for adulthood stressor exposure (see Fig 1). As the stress sensitization model suggests, it is possible that a high level of early adversity could be associated with enhanced stress-related personality traits, such as neuroticism and decreased positive traits such as extraversion, conscientiousness, agreeableness, and openness. In contrast, if the stress inoculation hypothesis holds, then levels of stress-related traits would be moderated by the levels of early adversity. Specifically, people experiencing a modest amount of adversity (compared to people experience a very low or high amount of adversity in the current samples) would show lower levels of neuroticism and higher levels of positive traits compared to those high and low in early adversity. The second aim was to examine the moderating role of early adversity on the associations between stress experiences (including stressor exposure and perceived stress) and personality traits in adulthood. Specifically, we examined whether the associations between exposure to stressors/perceived stress and the Big Five personality traits vary on the basis of early adversity (see Figs 2 & 3). According to previous research, stress experiences in adulthood were shown to have positive relations to stress-related traits such as neuroticism while displayed negative relations to positive traits such as extraversion, agreeableness, conscientiousness, and openness [6]. Thus, if the stress sensitization hypothesis holds, as early adversity increases, personality traits are more likely to be influenced by adulthood stress experiences such that stress (stressor exposure and perceived stress) in adulthood was expected to show stronger positive associations with neuroticism and stronger negative associations with positive traits in individuals reported higher levels of early adversity. However, if the stress inoculation model applies to the stress-personality relations, weaker associations were expected to be observed between adulthood stress and personality traits among individuals reported moderate levels of early adversity. Based on availability of the data of personality, exposure to stressors, and perceived stress, the first sample we used examined the above-mentioned research questions using data from the Health and Retirement Study (HRS). In the second sample, we tested the research questions using a middle-aged sample from the Midlife in the United States Survey (MIDUS). The HRS contained data for different types of adulthood stressor exposure (e.g., traumatic stressors, stressful life events, and chronic stressors). Given that previous studies have suggested that stress in different dimensions (e.g., traumatic events vs. stressful events, chronic stressors vs. episodic stressors) may demonstrate differential relations with other constructs [46], in the HRS sample, we tested the moderating effects of early adversity on the associations between different types of adulthood stressor exposure and personality traits.

In the present study, the level of early adversity was operationalized as the number of adverse events reported by participants. Also, throughout the paper, we used stressor exposure and perceived stress to refer to stress experienced in adulthood.

## Method

### Participants

#### HRS

The data used in Sample 1 were drawn from the Health and Retirement Study (HRS). Aiming at providing data for multidisciplinary investigations on important questions about the challenges and opportunities of aging, HRS surveys a nationally representative longitudinal sample of approximately 20,000 Americans aged 50 and older [47]. Participants in the HRS were assessed every 2 years on health, employment, and wealth. Starting in 2006, participants completed a psychosocial questionnaire that included measures of personality traits and the experience of stress. Half of the HRS participants completed the psychosocial questionnaire in 2006, and the other half completed it in 2008. In the current study, the two samples assessed in 2006 and 2008 were combined. 6097 participants (61.8% female) who provided data for at least 90% of items of the early adversity measure were retained in the current study. The mean age of the sample was 65.04 (SD = 8.89). Participants reported an average of 13.25 years of education. About 85.9% of the participants were self-identified as White or Caucasian, 10.6% as African American, and 3.5% as other ethnicities. Information about the two subsamples (surveyed in 2006 and 2008) is presented in S1 Fig and S1 Table.

#### MIDUS

The data used in Sample 2 were drawn from the Midlife in the United States Survey (MIDUS I and II) [48]. The survey data were collected from a nationally representative sample with the participants completing a 30-min telephone interview and self-administered questionnaires. A total of 4586 (53.8% female) participants who had data on 90% of the items for early adversity were included in the present study. Participants had a mean age of 55.64 (SD = 12.42). 65.7% of the sample had at least 1 to 2 years of college education.

### Measures

#### Early adversity

*HRS*. Items of measures of early adversity are listed in S1 Table. The initial selection of early adversity items included all items tapping into experiences before 16 or 18. Then 3 researchers independently rated each item according to the definition of early adversity (items measure experiences that may acquire significant adaptation by an average child and that represent a deviation from the expectable environment [10]). Four items measured in 2006 and 2008 (from the core HRS survey) and 6 items measured in the Life History Mail Survey (a questionnaire mailed to subsamples of the HRS participants in 2015 and 2017 to inquiry about residential history, education history, and other important childhood and family events) were used to index exposure to adversity early in life based on agreement among raters. The selected items included measures of family-related and school-related adversities. Participants were asked to check the occurrence of the adverse events on a binary scale based on their experiences before they were 16 or 18 years old (6 items measured adversities experienced before Age 16 and 4 items measured adversities occurred before Age 18, see S2 Table for details). As the number of participants who endorsed 6 to 10 items of early adversity was small in each level (between 1 and 55), endorsements between 6 and 10 were combined as “experienced 6 or more of the early adversity” to avoid extreme skewness in data. Cronbach alpha for the measure of early adversity was .60.

*MIDUS*. Items of early adversity are listed in S2 Table. As described for the HRS sample, based on rating agreement among raters, 27 items measured in MIDUS 1 were used to index early adversity, including measures of financial difficulties, parent’s death, parents’ divorce, parents physical and mental health problems, and exposure to early maltreatment (assessed via phone interview). The questions assessing childhood abuse (assessed via self-administered questionnaire) were taken from a revised version of the Conflict Tactics Scale [49]. Participants were presented with a list of emotional, moderate and severe physical abusive behaviors and were instructed to indicate the frequency on a 4-point scale (1 = often; 2 = sometimes; 3 = rarely; 4 = never) of each type of abuse in reference to mother, father, sister, brother, and anyone else. The abuse items were dichotomized such that “often” and “sometimes” were combined as “1” and “rarely” and “never” were combined as “0”. As the number of participants who endorsed 11 to 18 (the maximum number of endorsement) items of early adversity was small in each level (between 3 and 112), endorsements between 11 and 18 were combined as “experienced 11 or more of the early adversity”.

#### Personality

*HRS*. The Big Five personality traits were measured using the MIDUS Big Five Adjectival scale [50]. A total of 26 adjectives were used to assess neuroticism, extraversion, agreeableness, conscientiousness, and openness. Each adjective was rated on a four-point scale with 1 as “a lot” and 4 as “not at all”. Cronbach alphas for the five personality traits in the present sample were as follows: .73 for neuroticism, .75 for extraversion, .78 for agreeableness, .66 for conscientiousness, and .79 for openness. Personality items used in the current study are displayed in S3 Table.

*MIDUS*. The Big Five personality traits were measured by the Midlife Development Inventory (MIDI) [51] in MIDUS II via self-administered surveys. The MIDI personality inventory contains 25 adjectives that assess neuroticism, conscientiousness, extraversion, agreeableness, and openness. The items were rated on a 4-point scale from 1 “not at all” to 4 “a lot”. Cronbach alphas for each trait were as follows: .74 for neuroticism, .76 for extraversion, .80 for agreeableness, .58 for conscientiousness, and .77 for openness. Items used of assess personality traits in can be found in S3 Table.

#### Adulthood exposure to stressors

Across both samples, items that measure events that individuals may appraise as overwhelming, uncontrollable, and/or unpredictable. **HRS. Traumatic events.** To measure the measure of traumatic events experienced after age 18, participants were instructed to indicate whether each of the 7 traumatic events (including experiences of disasters, physical attack, and death or illness of close others) occurred at any point in their life. If the event did happen, participants also indicated the age they experienced it. Only traumatic events occurred after age 18 were included in the analyses. **Stressful life events.** To measure of stressful life events, participants were instructed to check whether they experienced each of the 5 major stressful life events (including measures of events such as work and family/household related stressful experiences) at some point in the past 5 years on a binary scale. **Ongoing chronic stressors.** The measure of ongoing chronic stressors was available for participants assessed in 2006 as assessed by 7 items capturing participants’ subjective experiences of ongoing stressors in different areas of life (including measures of chronic work, family, and financial difficulties). Each item was rated on a 4-point scale (1 = no, didn’t happen; 2 = yes, but not upsetting; 3 = yes, somewhat upsetting; 4 = yes, very upsetting). To assess the objective experience of the stressors, each item of the ongoing chronic stressors was recoded to indicate the occurrence of each stressor (2 to 4 on the original scale were combined). Items used to assess adulthood stressor exposure are shown in S4 Table.

*MIDUS*. Exposure to stressors were measured by 15 items assessing occurrence of traumatic events in MIDUS II via self-administered surveys. For each of the events, participants were instructed to indicate whether the event ever occurred or not. For the event that did happen, participants indicated the age at which they experienced it. Only events experienced after age 18 were included in the analyses. Items used to assess stressor exposure are presented in S4 Table.

#### Perceived stress

*HRS*. Based on a recent review [52] for peer-reviewed publications that used the HRS to examine associations between psychosocial stress and different outcomes, items used as stress measures were included in the initial selection. Then two researchers independently rated each item based on the definition of perceived stress (items measure the extent to which individuals appraise certain experiences as overwhelming, uncontrollable, and/or unpredictable), and items were retained according to agreement between raters. According to results of the exploratory factor analysis (details can be found at https://osf.io/x4pe3/?view_only=af103c0e8cd648c69e042b825b2b0a7e), the items measured perceived stress covered perceptions of stress in varying settings and aspects of daily life including perceived discrimination (5 items), perceived job stress (3 items), perceived family stress (7 items), perceived interpersonal stress (12 items), and perceived neighborhood stress (8 items). Cronbach alphas for perceived stress was .87. All the perceived stress items are presented in S5 Table.

*MIDUS*. Because a standard measure of perceived stress was not available in the MIDUS data, a measure of perceived stress was developed out of the items in the survey (see Luo et al., 2017 for details) [6]. Seven items chosen from different sections (6 from self-administered surveys and 1 from phone interview) of the Midlife Development Inventory (MIDI) [53] that tapped into participants’ perceived stress in work, financial situations, relationships, and life in general were used. In an MTurk sample that provided data on both the 7-item perceived stress scale and the Perceived Stress Scale (PSS) [54], the 7-item stress scale showed item-total correlations ranging from .30 to .74, the average inter-item correlation of .37, and alpha reliability of .79. Also, the correlation between the total score of the 7 items and the PSS total score was .73 in the MTurk sample. In the current MIDUS sample, Cronbach alphas was .73. Perceived stress items are listed in S5 Table.

### Statistical analysis

All the analyses were conducted using Mplus 8.0 [55]. The scripts for the analyses that are described can be found at https://osf.io/x4pe3/?view_only=af103c0e8cd648c69e042b825b2b0a7e. Full information maximum likelihood (FIML) was used for estimation due to missing data across the time points. Across the two samples, composite scores were calculated for early adversity, adulthood exposure to traumatic events, stressful life events (the HRS only), and ongoing chronic stressors (the HRS only) by adding up endorsement on each item. The latent variables for each of the Big Five personality traits and perceived stress were specified. The 4 neuroticism items, 5 extraversion items, 5 agreeableness items, 5 conscientiousness items (4 items in the MIDUS sample), and 7 openness items were used as manifest indicators for the latent traits of neuroticism, extraversion, agreeableness, conscientiousness, openness. For the HRS sample, the composite scores of the 5 perceived stress domains were used as manifest indicators for the latent factor of perceived stress, and the 7 perceived stress items were used as manifest indicators in the MIDUS sample. For model simplicity, separate analyses were conducted to each of the Big Five personality traits.

The stress sensitization hypothesis implies a linear relationship between early adversity and outcome variables, while the stress inoculation hypothesis suggests a possible quadratic relationship early adversity and outcomes such that beneficial effects may be observed among individuals with moderate exposure to adversity early in life. To investigate the relationship between early adversity and the level of personality traits, for both samples, we first regressed the latent variables for each of the five personality traits on the composite score of early adversity and the squared early adversity score. Age and gender were controlled in all the models. As early adversity and adulthood stressor exposure have some nontrivial overlap (see Tables 1 and 2 for the correlations between early adversity and adulthood stressor exposure in the HRS and the MIDUS samples), we also ran another set of regression models by adding adulthood stressors (indexed by the composite scores of traumatic events and stressful life events in the HRS and the composite scores of traumatic events in the MIDUS) as control variables. Due to missing data in ongoing chronic stressors for a subsample of participants, in the HRS, the composite score of ongoing chronic stressors was not included as a covariate (We conducted analyses that included ongoing chronic stressors measured in 2010 and 2012 (N = 5532)) as a covariate, and the results pattern remained the same).

**Table 1:** Correlations among manifest variables in the HRS sample.

	EA	N	E	A	C	O	TE	SLE	COS	PS
EA	-	.08**	-.02	-.03*	-.09**	-.02	.11**	.09**	.12**	.13**
N		-	-.21**	-.11**	-.23**	-.21**	.01	.12**	.29**	.41**
E			-	.54**	.36**	.52**	.02	-.04**	-.15**	-.15**
A				-	.40**	.38**	.02	-.02	-.04*	-.18**
C					-	.43**	-.01	-.02	-.15**	-.23**
O						-	.06**	.03*	-.07**	-.08**
TE							-	.07**	.22**	.07**
SLE								-	.23**	.18**
COS									-	.44**
PS										-

*Note*.

*p < .05

**p < .01. EA = early adversity; N = neuroticism; E = extraversion; A = agreeableness; C = conscientiousness; O = openness; TE = traumatic events; SLE = stressful life events; COS = chronic ongoing stressors; PS = perceived stress.

**Table 2:** Correlations among manifest variables in the MIDUS sample.

	EA	N	E	A	C	O	TE	PS
EA	-	.14**	-.01	-.04*	-.07**	.01	.19**	.13**
N		-	-.20**	-.12**	-.20**	-.22**	.09**	.42**
E			-	.51**	.26**	.51**	-.05*	-.33**
A				-	.27**	.33**	-.01	-.17**
C					-	.28**	-.09**	-.31**
O						-	.05*	-.25**
TE							-	.20**
PS								-

*Note*.

*p < .05

**p < .01. EA = early adversity; N = neuroticism; E = extraversion; A = agreeableness; C = conscientiousness; O = openness; TE = traumatic events; PS = perceived stress.

Then the moderating effects of early adversity on the covariance between stress experiences and personality traits were tested in a series of models using the moderated nonlinear factor analysis (MNLFA) model [56]. Originally developed for evaluating differential item functioning, the MNLFA model permits model parameters, such as variances, covariances, and factor loadings, to differ as a function of multiple individual characteristics (e.g., age, gender, educational level). MNLFA is more flexible than multiple group analysis because it allows researchers to examine both categorical and continuous moderators (e.g., early adversity). Using the MNLFA model, we tested whether early adversity moderated the associations between the adulthood experiences of stress and personality traits in the two samples. Specifically, the covariances between different types of adulthood stressors exposure/perceived stress and personality traits were allowed to vary on the basis of early adversity. Each of the models included the composite score of early adversity and the squared early adversity score in moderation of the two focal constructs, variance of the two constructs, and moderation of the covariance of the two constructs. Age and gender were controlled in all models. The moderating effects of early adversity and the quadratic term of early adversity on the covariances were the focus in this step of the analyses. Common model fit indices like CFI, TLI, and RMSEA for MNLFA were not available in Mplus by the time we performed the analysis.

## Results

Tables 1 and 2 display the correlations of manifest variables included in the analyses in the HRS and the MIDUS samples. As shown in the table, across the two samples, early adversity exhibited significant positive correlations with neuroticism, traumatic events, stressful life events (HRS only), chronic ongoing stressors (HRS only), and perceived stress and significant negative correlations with agreeableness and conscientiousness. Traumatic events were positively related to openness in the HRS and positively associated with neuroticism and openness in the MIDUS. In the HRS, stressful life events were positively associated with neuroticism and openness, and negatively associated with extraversion. Chronic ongoing stressors were positively linked to neuroticism and negatively linked to extraversion, agreeableness, conscientiousness, and openness. Finally, in both samples, neuroticism showed significant positive correlations with perceived stress, while extraversion, agreeableness, conscientiousness, and openness displayed significant negative associations with perceived stress.

### Early adversity and personality

**Fig 4:**
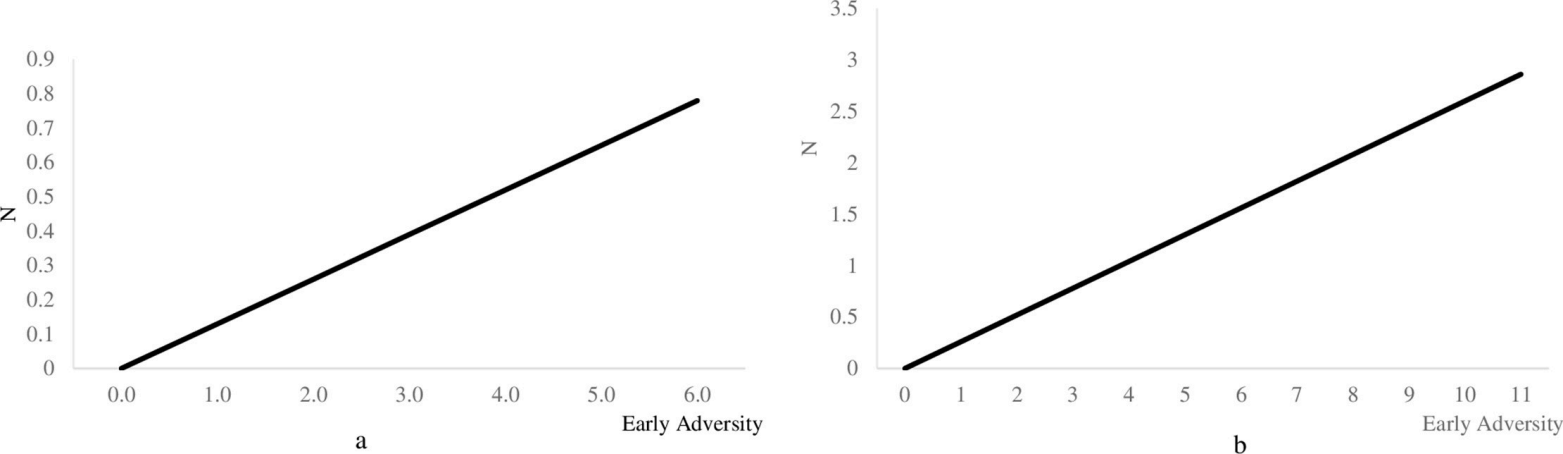
a & b. The effects of early adversity on neuroticism after controlling for adulthood stressors exposure in the HRS and the MIDUS samples. In HRS, adulthood stressors exposure included traumatic events and stressful life events. Traumatic events included experiences of disasters, physical attack, and death or illness of close others. Stressful life events included measures of events such as work and family/household related stressful experiences. In MIDUS, adulthood stressors exposure included measures related to job, family, financial, and other aspects of traumatic experiences in life. N = Neuroticism.

**Fig 5:**
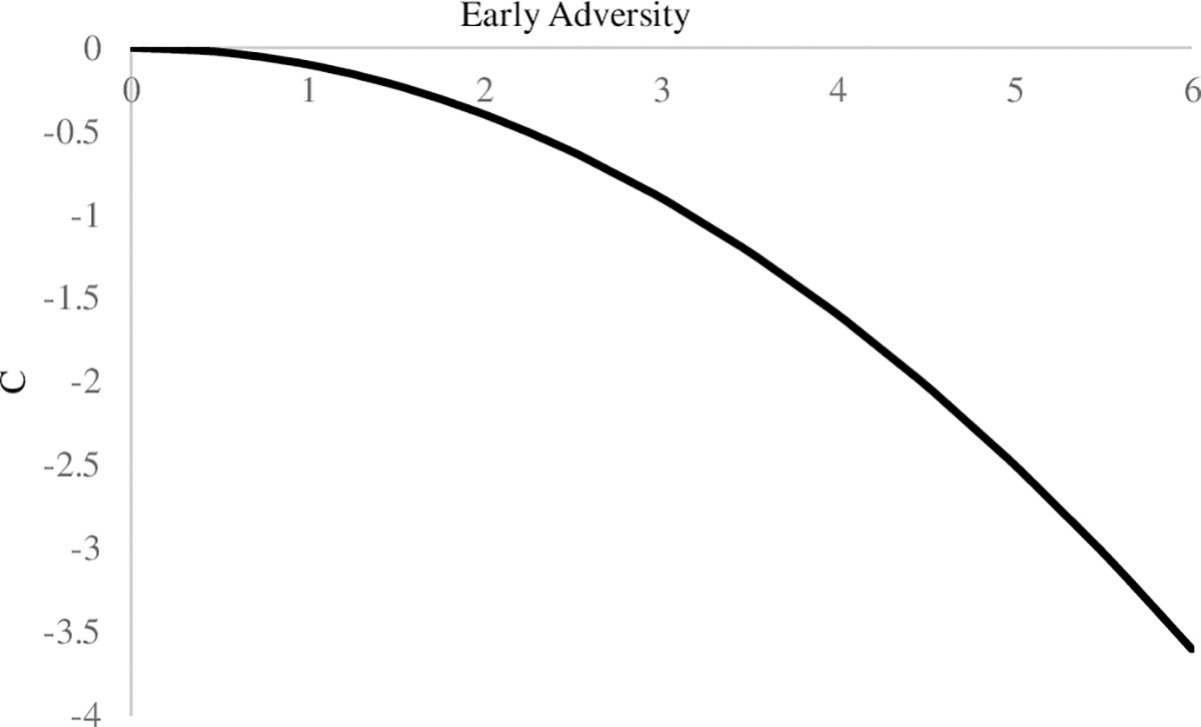
The effects of early adversity on conscientiousness after controlling for adulthood stressors exposure in the HRS sample. Adulthood stressors exposure included traumatic events and stressful life events. Traumatic events included experiences of disasters, physical attack, and death or illness of close others. Stressful life events included measures of events such as work and family/household related stressful experiences. C = Conscientiousness.

We first tested the linear and quadratic effects of early adversity on the Big Five personality traits. The results are presented in Tables 3 and 4 for the HRS and the MIDUS samples. As shown in the tables, in both samples, early adversity displayed significant linear, but not quadratic, associations with neuroticism (*β* = .14, *p* < .001 in HRS and *β* = .18, *p* = .007 in MIDUS). Early adversity displayed significant quadratic associations with agreeableness (*β* = -.07, *p* = .048) and conscientiousness (*β* = -.09, *p* = .024) in HRS but not MIDUS. After controlling for adulthood stressors exposure, consistently, in both samples, the linear relation between early adversity and neuroticism (*β* = .13, *p* < .001 in HRS and *β* = .26, *p* = .003 in MIDUS) remained significant. Also, in HRS, the quadratic relation between early adversity and conscientiousness (*β* = -.10, *p* = .010) remained significant after considering the effects of adulthood stressors exposure. As shown in Fig 4A & 4B, within the range of early adversity assessed in the two samples, individuals exhibited higher levels of neuroticism as early adversity increased. Fig 5 depicts that in the HRS sample, individuals demonstrated lower levels of conscientiousness as early adversity increased, while the level of conscientiousness decreased in an accelerated way as the score of early adversity increased. The pattern in both samples was aligned with the stress sensitization model such that increases in early adversity were associated with negative development of personality traits after controlling for stressor exposure in adulthood.

**Table 3:** Standardized estimates of the linear and quadratic effects of early adversity on personality traits with and without controlling for adulthood stressors exposure in the HRS sample.

	Without controlling for stressors exposure				Controlling for stressors exposure			
	β_EA_	p-value	β_EA_^2^	p-value	β_EA_	p-value	β_EA_^2^	p-value
Neuroticism	.14	< .001	-.07	.074	.13	< .001	-.06	.097
Extraversion	.02	.561	-.04	.283	.03	.458	-.05	.209
Agreeableness	.07	.072	-.07	.048	.07	.070	-.07	.062
Conscientiousness	-.01	.742	-.09	.024	-.004	.913	-.10	.010
Openness	.004	.911	-.02	.538	.01	.785	-.04	.254

*Note*. Adulthood stressors exposure included traumatic events and stressful life events. Traumatic events included experiences of disasters, physical attack, and death or illness of close others. Stressful life events included measures of events such as work and family/household related stressful experiences. EA = early adversity.

**Table 4:** Standardized estimates of the linear and quadratic effects of early adversity on personality traits with and without controlling for adulthood stressors exposure in the MIDUS sample.

	Without controlling for stressors exposure				Controlling for stressors exposure			
	β_EA_	p-value	β_EA_^2^	p-value	β_EA_	p-value	β_EA_^2^	p-value
Neuroticism	.18	.007	-.05	.472	.26	.003	-.14	.107
Extraversion	.13	.060	-.12	.090	.04	.646	-.02	.788
Agreeableness	.05	.463	-.03	.631	.08	.336	-.04	.651
Conscientiousness	-.06	.401	-.01	.926	.11	.262	-.15	.124
Openness	.05	.518	-.02	.726	.12	.186	-.12	.193

*Note*. Adulthood stressors exposure included traumatic events, including measures related to job, family, financial, and other aspects of traumatic experiences in life. EA = early adversity.

### Early adversity and associations between stress and personality

**Fig 6:**
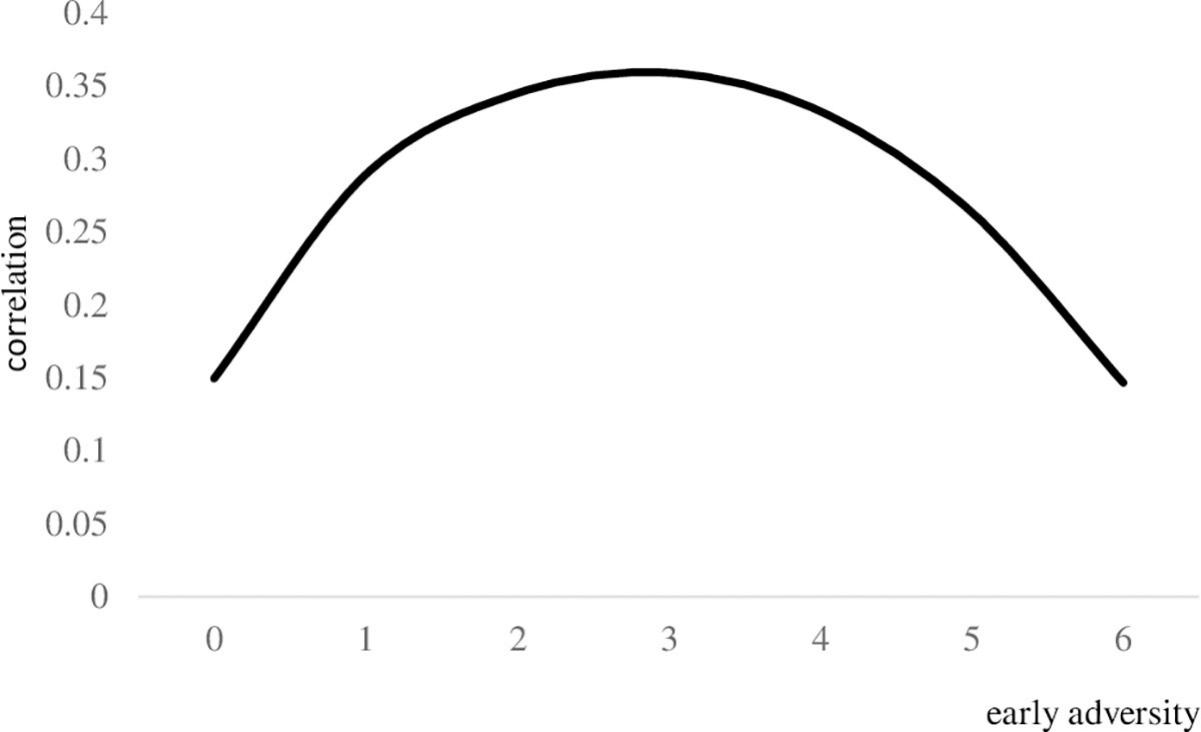
The correlations between ongoing chronic stressors and neuroticism on the basis of early adversity in the HRS sample. Ongoing chronic stressors included measures of chronic work, family, and financial difficulties.

Tables 5 and 6 display estimations for the moderating effects of early adversity on the associations between adulthood stress and the Big Five personality traits in the HRS and MIDUS samples. Given that 20 tests were conducted in HRS in total, we used Bonferroni corrected alpha (p ≤ .003) which was adjusted to the number of hypotheses tested to define significance. Consistently, for MIDUS, we used Bonferroni corrected alpha (p ≤ .005) which was adjusted to the number of hypotheses (N = 10) tested to define significance As shown in the table, in HRS, early adversity only displayed significant linear and quadratic moderating effects on the associations between ongoing chronic stressors and neuroticism. Contrary to what is suggested by the stress inoculation hypothesis, we found an inverted U-shape (Fig 6) such that the level of neuroticism was more strongly tied to the experience of ongoing chronic stressors among those who experienced a moderate amount of adversity early in life. Across the two samples, the associations between traumatic events, stressful life events (HRS only), perceived stress and the level of personality traits were not moderated by early adversity.

**Table 5:** Parameter estimates of the linear and quadratic moderating effects of early adversity on the covariances between different types of adulthood stressors exposure and adulthood perceived stress and personality traits in the HRS sample.

	EA	p-value	EA^2^	p-value
Traumatic events				
Neuroticism	-.004	.876	.003	.659
Extraversion	.01	.775	.002	.722
Agreeableness	-.003	.918	.001	.815
Conscientiousness	.004	.878	0	.978
Openness	-.002	.935	-.001	.892
Stressful life events				
Neuroticism	-.03	.394	.002	.828
Extraversion	.05	.150	-.003	.702
Agreeableness	.04	.305	-.004	.557
Conscientiousness	.04	.341	-.01	.436
Openness	-.01	.793	.01	.406
Ongoing chronic stressors				
Neuroticism	.11	.001	-.01	.121
Extraversion	-.06	.098	.004	.551
Agreeableness	-.02	.621	-.001	.857
Conscientiousness	-.06	.117	.01	.413
Openness	-.02	.481	0	.990
Perceived stress				
Neuroticism	-.10	.011	.02	.043
Extraversion	.06	.095	-.01	.416
Agreeableness	.04	.199	-.01	.396
Conscientiousness	.07	.046	-.004	.587
Openness	.04	.267	-.003	.674

*Note*. EA = estimation of the linear effects of early adversity; EA^2^ = estimation of the quadratic effects of early adversity. Traumatic events included experiences of disasters, physical attack, and death or illness of close others. Stressful life events included measures of events such as work and family/household related stressful experiences. Ongoing chronic stressors included measures of chronic work, family, and financial difficulties.

**Table 6:** Parameter estimates of the linear and quadratic moderating effects of early adversity on the covariances between adulthood stressors exposure and adulthood perceived stress and personality traits in the MIDUS sample.

	Level					
	EA		p-value	EA^2^		p-value
Traumatic events						
Neuroticism	-.001		.980	0		.868
Extraversion	.03		.298	-.003		.285
Agreeableness	.02		.460	-.002		.524
Conscientiousness	.01		.835	0		.994
Openness	.01		.669	0		.867
Perceived stress						
Neuroticism	-.03		.362	.003		.315
Extraversion	-.02		.604	.001		.836
Agreeableness	.02		.399	-.002		.413
Conscientiousness	-.04		.264	.003		.310
Openness	.001		.978	0		.970

*Note*. Traumatic events included measures related to job, family, financial, and other aspects of traumatic experiences in life. EA = estimation of the linear effects of early adversity; EA^2^ = estimation of the quadratic effects of early adversity.

## Discussion

Using two samples from large longitudinal panel studies, the HRS and MIDUS, the present research examined the linear and quadratic relations between early adversity and the Big Five personality traits, as well as the moderating effects of early adversity on the relationships between stress experiences and personality traits in adulthood. According to the results, early adversity demonstrated positive associations with neuroticism, even after accounting for the effects of exposure to stressors in adulthood. Results from HRS indicated the moderating role of early adversity in the association between ongoing chronic stressors and neuroticism. The results from both linear and curvilinear models were in line with the stress sensitization model, and no support was found for the stress inoculation hypothesis.

In both samples, early adversity did not show curvilinear relationships with the Big Five personality traits as predicted by the stress inoculation hypothesis. Instead, early adversity consistently demonstrated positive linear associations with neuroticism. The linear relations to neuroticism remained significant after controlling for the effects of adulthood stressor exposure. In general, the results were consistent with the stress sensitization hypothesis as higher levels of early adversity showed positive associations with the levels of neuroticism. The sociogenomic model of personality suggests a dynamic process of the development of personality traits in which states mediate the relationship between the environment and personality trait development [57]. Specifically, the model proposes that experiences, such as early adversity, act largely upon states (the moment-to-moment fluctuations in thoughts, feelings, and behaviors that reflect immediate response to changes in one’s environment [57]), and the changed states will result in the development of personality traits after being internalized, automatized, and generalized. Individuals with exposure to early adversity have been shown to be more likely to interpret ambiguous situations as threatening, leading to increased levels of stress perception [58]. Also, it has been suggested that individuals who experienced adversity early in life tend to display attention biases in a way that they are more likely to attend to threatening cues and allocate more attentional resources to threatening stimuli [59, 60]. Thus, it is possible that individuals who were exposed to early adversity were more likely to experience states, such as negative moods, which resulted in higher levels of neuroticism over time.

Also, previous research has suggested that early adversity is likely to influence adulthood outcomes through environmental continuity such that the later exposure largely accounts for any variance associated with early adversity [35, 61]. Results in the current study indicated that even after taking the influences of adulthood stressors exposure into account, early adversity still exert incremental influences on neuroticism. Moreover, most of the studies testing the influences of early adversity on personality focused on the periods of adolescence or young adulthood. Our study extended the findings to show that individuals exposed to adversity early in life tended to have a higher level of neuroticism even in middle ages and older ages. However, the unique effects of early adversity were not observed on other personality traits. Given that neuroticism is the trait domain that most closely aligned with stress experiences among the Big Five, it is expected that the development of neuroticism is more strongly linked to early experiences of adversity than other traits.

Results from the HRS sample suggested the moderating effects of early adversity on the associations between ongoing chronic stressors and neuroticism. The link between ongoing chronic stressors and neuroticism varied on the basis of early adversity. Contrary to the stress inoculation hypothesis, inverted U-shaped relations were observed such that individuals with a moderate exposure to early adversity showed stronger associations between ongoing chronic stressors and neuroticism. Therefore, rather than protecting individuals from the impacts of stressors exposure, individuals with moderate early adversity were more likely to have their neuroticism tied to the experiences of ongoing chronic stressors. However, the measure of ongoing chronic stressors was available only in the older but not the middle-age sample. Future replication is needed with different age groups.

According to the results in HRS, early adversity demonstrated moderating effects on the associations between ongoing chronic stressors and personality traits, but not other dimensions of stress (e.g., traumatic event, stressful life events, and perceived stress). The nonsignificant effects on traumatic events and perceived stress were replicated in MIDUS. Previous studies have highlighted the importance of examining stress in different dimensions [62] as the adjustment may differ according to stress types. The effects of early adversity may vary depending on the dimension of adulthood stress examined. Compared to acute stressors, exposure to ongoing chronic stressors is more likely to result in a continuous and persistent state, the effects of which may accumulate to lead to changes in personality traits. Thus, the effects of early adversity may be more salient in the relationship between ongoing stressors and personality traits. Although future replication is needed, results of the current study suggests the necessity to take individual differences in early life experiences into consideration when examining personality development in adulthood. The present results provided some evidence that how stress-related traits (e.g., neuroticism) develop in response to stress experiences in adulthood, especially to chronic stressful conditions, may vary as a function of individuals’ early adverse experiences. However, according to the current findings, the stress inoculation model may not apply to personality traits in middle or late adulthood.

According to the current results, signs were found such that early adversity may play a role in the development of conscientiousness in late but not middle adulthood. In the HRS sample, increases in early adversity were associated with accelerating decreases in conscientiousness after accounting for adulthood exposure to stressors. It is possible that early adversity displays differential effects on the development of conscientiousness in samples at different life stages. Results in both samples did not provide support for the stress inoculation hypothesis in personality development. Further investigation is needed on the role of early adversity in the development of conscientiousness over the lifespan.

Despite several strengths (e.g., the use of multiple representative longitudinal samples), the current study is still limited in several aspects. First, the assessment of early adversity in the two samples was not exhaustive. Previous research has suggested that adverse events in childhood tend to co-occur with each other [8]. Individuals who experienced one form of adversity are likely to also have experienced other adversities. It is possible that the curvilinear relationships between early adversity and other parameters in the models are more salient when a wider range of early adversity was assessed. Also, the potential beneficial effects of moderate early adversity may be offset by more severe ones when a comprehensive assessment is conducted [63]. Moreover, there is one caveat to the measure of early adversity in both samples that the items assessed participants’ experiences before the age of 16 or 18. However, studies about early adversity usually measured experiences earlier than that assessed in the current study. It would be more appropriate to interpret the early adversity assessed in the current study as the adversity experienced prior to adulthood.

Also, the current research used the sum scores from checklist measures as an indicator of the level of early adversity. However, such a method did not take the severity of individual stressor into account. The presence of childhood abuse was measured in the MIDUS sample, but the severity of the experiences was not evaluated (e.g., moderate physical abuse vs. severe physical abuse). Although there is no consensus on what should be viewed as “a moderate level of adversity” in the stress inoculation hypothesis, the importance of differentiating the severity of individual stressor has been agreed upon [64, 65]. Future studies should employ interview approaches to assess early adversity so that different factors, such as the frequency, persistence, recurrence, and other contextual information pertaining to the stressor, can be incorporated to determine the severity of each stressor.

Third, the effects of different types of early adversity were not tested in the current study. Future research should examine whether different types of early adversity, such physical abuse, sexual abuse, and financial difficulties, exert differential influences on personality and the associations between stress experiences and personality in adulthood.

Finally, the current findings should be interpreted with caution that early adversity was measured only in a retrospective way in both samples. It has been suggested that the relationship between early adversity and other outcomes in the adulthood can be biased by retrospective report of childhood adversity. Retrospective assessment of early adversity may result in under-reporting of the adversity due to recall failure [66], and on the other hand, the assessment may also be biased by the participants’ current mental state [67]. According to a meta-analytic review, agreement between retrospective and prospective measure of childhood maltreatment was relatively low [68], suggesting that childhood maltreatment assessed in the two manners may not be used interchangeably. Mixed findings have been reported on the comparison of findings from retrospective and prospective assessment of early adversity. For example, one study found a significant association between retrospectively, but not prospectively, measured childhood maltreatment and drug misuse in young adulthood, suggesting that the association between childhood maltreatment and mental health outcomes in adulthood may be spurious due to recall bias [69]. However, no difference in the strength of the associations between childhood maltreatment and mental disorders in young adulthood was reported in another study [70]. Using both prospective and retrospective measures, a study reported that the associations found between retrospective measures of childhood adversity and negative life outcomes were confirmed by findings from prospective records [71]. However, according to the results, the impact of early adversity was underestimated for objectively assessed outcomes while overestimated for self-reported outcomes among individuals with high levels of neurotic and agreeable dispositions. Therefore, based on findings from previous studies, it is possible that the relationship between early adversity and adulthood personality traits, as well as the associations between adulthood stress experiences and personality, may display a different pattern from the current findings when prospective measures of early adversity are used. Meanwhile, according to previous research, it is possible that retrospective report of early adversity can be biased by adulthood personality. For example, individuals high on neuroticism are likely to have a more negative recall of early life experiences than others, whereas those high on agreeableness are prone to a more positive recall. If such biases exist, adopting retrospective measure of early adversity would make it difficult to detect the inoculating effects, with the sensitizing effects more likely to be present. Taken together, results from the current study can only be viewed as a preliminary evidence for the inoculating vs. sensitizing effects of early adversity on the covariance between adulthood stress experiences and personality traits. Future study should examine the relations among early adversity, adulthood stress experiences and personality traits using both retrospective and prospective measures and investigate their agreement in results.

In sum, the current study tested the stress sensitization vs. stress inoculation hypotheses by examining the associations between early adversity and adulthood personality traits, as well as the moderating effects of early adversity on the associations between personality traits and stress experiences in adulthood. Results in the present research were more in line with the stress sensitization model in personality development in mid and late adulthood. No support was found for the stress inoculation model in personality development. Future studies are needed to detect types of adulthood stress and the sensitive ages that are pertinent to effects of early adversity on the associations between adulthood stress and personality traits.

## Supporting information

S1 Fig Demographic information for the two subsamples in the HRS. (TIF)

S1 Table Demographic information for the two subsamples in HRS. (DOCX)

S2 Table Items used to measure early adversity in the HRS and the MIDUS samples. (DOCX)

S3 Table Items used to measure the Big Five personality traits in the HRS and the MIDUS samples. (DOCX)

S4 Table Items used to measure exposure to stressors in adulthood in the HRS and the MIDUS samples. (DOCX)

S5 Table Items used to measure perceived stress in the HRS and the MIDUS samples. (DOCX)
