# Supplementary figures and images for: Sensitization or inoculation: Investigating the effects of early adversity on personality traits and stress experiences in adulthood
Source: PLoS One. 2021 Apr 1;16(4):e0248822. doi: 10.1371/journal.pone.0248822 (PMC8016298; doi:10.1371/journal.pone.0248822)

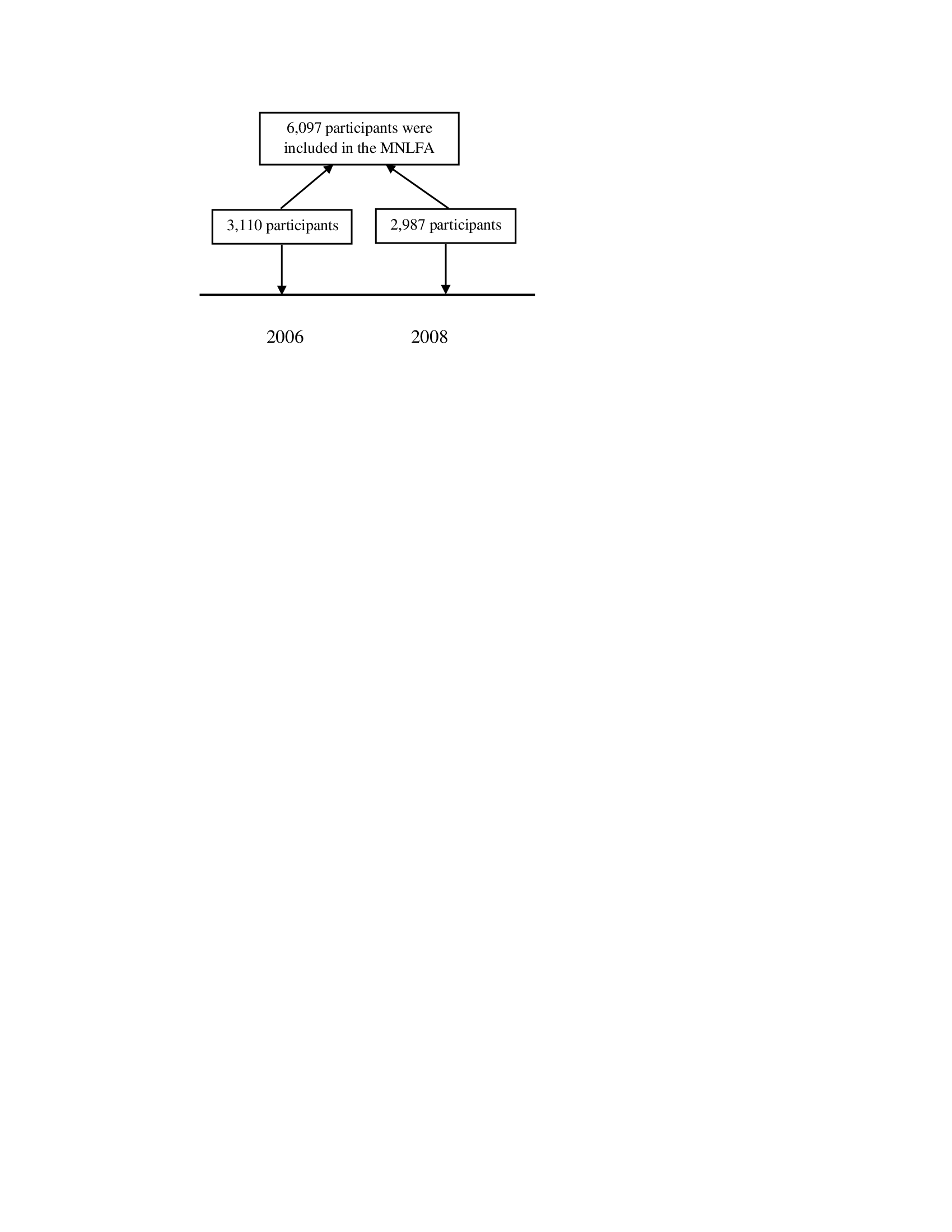

Supplement: S1 Fig — (TIF) [file pone.0248822.s001.tif]
