# Supplementary material for: Sensitization or inoculation: Investigating the effects of early adversity on personality traits and stress experiences in adulthood
Source: PLoS One. 2021 Apr 1;16(4):e0248822. doi: 10.1371/journal.pone.0248822 (PMC8016298; doi:10.1371/journal.pone.0248822)
Supplement: S1 Table — (DOCX) [file pone.0248822.s002.docx]

**S1 Table. Demographic information for the two subsamples in HRS.**

|  | Mean age (SD) | Sex (% female) | Mean education |
| --- | --- | --- | --- |
| 2006 | 63.86 (8.87) | 61.2% | 13.34 |
| 2008 | 66.26 (8.74) | 62.4% | 13.15 |
