# Supplementary material for: Sensitization or inoculation: Investigating the effects of early adversity on personality traits and stress experiences in adulthood
Source: PLoS One. 2021 Apr 1;16(4):e0248822. doi: 10.1371/journal.pone.0248822 (PMC8016298; doi:10.1371/journal.pone.0248822)
Supplement: S2 Table — (DOCX) [file pone.0248822.s003.docx]

**S2 Table. Items used to measure early adversity in the HRS and the MIDUS samples.**

| **HRS** |  |
| --- | --- |
| Before you were 18 years old, did you have to do a year of school over again? |  |
| Before you were 18 years old, were you ever in trouble with the police? |  |
| Before you were 18 years old, did either of your parents drink or use drugs so often that it caused problems in the family? |  |
| Before you were 18 years old, were you ever physically abused by either of your parents? |  |
| Before you were age 16, did you ever live in a children’s home or orphanage? |  |
| Before you were age 16, did your biological or adoptive parents separate or divorce? |  |
| Before you were age 16, did one or both of your biological or adoptive parents die? |  |
| Before you were age 16, did one or more of your siblings die? |  |
| Before you were age 16, were you ever separated from your mother for 6 months  or longer? |  |
| Before you were age 16, were you ever separated from your father for 6 months  or longer? |  |
| **MIDUS** |  |
| During your childhood and adolescence, was there ever a period of six months or more when your family was on welfare or ADC? |  |
| When you were growing up, was your family better off or worse off financially than the average family was at that time? |  |
| Mother died. |  |
| Father died. |  |
| Parents separated/divorced. |  |
| Parents never lived together/R never knew biol. mother/father. |  |
| Father engaged in alcohol or drug abuse. |  |
| Father had mental or emotional disability. |  |
| Father was harsh when punished you. |  |
| Mother engaged in alcohol or drug abuse. |  |
| Mother had mental or emotional disability. |  |
| Mother was harsh when punished you. |  |
| *Pre-question: Below, and on the next page, are three lists of things that happen to some children. After each list, please indicate how often your parents, siblings, or anyone else did things like this to you. (If a question does not apply because there was no such person in your family when you were growing up, circle 'does not apply'.* |  |
| **Emotional abuse:**  *LIST A: Insulted you or swore at you; Sulked or refused to talk to you; Stomped out of the room; Did or said something to spite you; Threatened to hit you; Smashed or kicked something in anger.* |  |
| During your childhood, how often did your mother, or the woman raised you, do any of the things on List A to you? |  |
| During your childhood, how often did your father, or the man raised you, do any of the things on List A to you? |  |
| During your childhood, how often did your brothers do any of the things on List A to you? |  |
| During your childhood, how often did your sisters do any of the things on List A to you? |  |
| During your childhood, how often did anybody else do any of the things on List A to you? |  |
| **Physical abuse:**  *LIST B: Pushed, grabbed, or shoved you; Slapped you; Threw something at you.* |  |
| During your childhood, how often did your mother, or the woman raised you, do any of the things on List B to you? |  |
| During your childhood, how often did your father, or the man raised you, do any of the things on List B to you? |  |
| During your childhood, how often did your brothers do any of the things on List B to you? |  |
| During your childhood, how often did your sisters do any of the things on List B to you? |  |
| During your childhood, how often did anybody else do any of the things on List B to you? |  |
| **Severe physical abuse:**  *LIST C: Kicked, bit, or hit you with a fist; Hit or tried to hit you with something; Beat you up; Choked you; Burned or scalded you.* |  |
| During your childhood, how often did your mother, or the woman raised you, do any of the things on List C to you? |  |
| During your childhood, how often did your father, or the man raised you, do any of the things on List C to you? |  |
| During your childhood, how often did your brothers do any of the things on List C to you? |  |
| During your childhood, how often did your sisters do any of the things on List C to you? |  |
| During your childhood, how often did anybody else do any of the things on List C to you? |  |
