# Supplementary material for: Sensitization or inoculation: Investigating the effects of early adversity on personality traits and stress experiences in adulthood
Source: PLoS One. 2021 Apr 1;16(4):e0248822. doi: 10.1371/journal.pone.0248822 (PMC8016298; doi:10.1371/journal.pone.0248822)
Supplement: S3 Table — (DOCX) [file pone.0248822.s004.docx]

**S3 Table. Items used to measure the Big Five personality traits in the HRS and the MIDUS samples.**

| **Neuroticism** | A lot/Some/A little/Not at all |
| --- | --- |
| Moody |  |
| Worrying |  |
| Nervous |  |
| Calm |  |
| **Extraversion** | A lot/Some/A little/Not at all |
| Outgoing |  |
| Friendly |  |
| Lively |  |
| Active |  |
| Talkative |  |
| **Agreeableness** | A lot/Some/A little/Not at all |
| Helpful |  |
| Warm |  |
| Caring |  |
| Softhearted |  |
| Sympathetic |  |
| **Conscientiousness** | A lot/Some/A little/Not at all |
| Organized |  |
| Responsible |  |
| Hardworking |  |
| Careless |  |
| Thorough* |  |
| **Openness** | A lot/Some/A little/Not at all |
| Creative |  |
| Imaginative |  |
| Intelligent |  |
| Curious |  |
| Broad-minded |  |
| Sophisticated |  |
| Adventurous |  |

*Note.* *Not available in MIDUS.
