# Supplementary material for: Sensitization or inoculation: Investigating the effects of early adversity on personality traits and stress experiences in adulthood
Source: PLoS One. 2021 Apr 1;16(4):e0248822. doi: 10.1371/journal.pone.0248822 (PMC8016298; doi:10.1371/journal.pone.0248822)
Supplement: S4 Table — (DOCX) [file pone.0248822.s005.docx]

**S4 Table. Items used to measure exposure to stressors in adulthood in the HRS and the MIDUS samples.**

| **HRS** |
| --- |
| Traumatic Events |
| Has a child of yours ever died? |
| Have you ever been in a major fire, flood, earthquake, or other natural disaster? |
| Have you ever fired a weapon in combat or been fired upon in combat? |
| Has your spouse, partner, or child ever been addicted to drugs or alcohol? |
| Were you the victim of a serious physical attack or assault? |
| Did you ever have a life-threatening illness or accident? |
| Did your spouse or a child of yours ever have a life-threatening illness or accident? |
| Stressful Life Events |
| Have you involuntarily lost a job for reasons other than retirement at any point in the past five years? |
| Have you been unemployed and looking for work for longer than 3 months at some point in the past five years? |
| Was anyone else in your household unemployed and looking for work for longer than 3 months in the past five years? |
| Have you moved to a worse residence or neighborhood in the past five years? |
| Were you robbed or did you have your home burglarized in the past five years? |
| Have you been the victim of fraud in the past five years? |
| Ongoing Chronic Stressors |
| Ongoing health problems (in yourself) |
| Ongoing physical or emotional problems (in spouse or child) |
| Ongoing problems with alcohol or drug use in family member |
| Ongoing difficulties at work |
| Ongoing financial strain |
| Ongoing housing problems |
| Ongoing problems in a close relationship |
| Helping at least one sick, limited, or frail family member or friend on a regular basis |
| **MIDUS** |
| Ever child died |
| Ever child experienced life-threatening |
| Ever lost home to fire/flood/etc |
| Ever physically assaulted |
| Ever sexually assaulted |
| Ever serious legal difficult/prison |
| Ever jail detention |
| Ever bankruptcy declared |
| Ever financial loss unrelated to work |
| Ever welfare |
| Ever SP engaged in infidelity |
| Ever significant in-law difficulties |
| Ever experienced combat |
| Ever fired from a job |
| Ever no job for long time |
