# Supplementary material for: Sensitization or inoculation: Investigating the effects of early adversity on personality traits and stress experiences in adulthood
Source: PLoS One. 2021 Apr 1;16(4):e0248822. doi: 10.1371/journal.pone.0248822 (PMC8016298; doi:10.1371/journal.pone.0248822)
Supplement: S5 Table — (DOCX) [file pone.0248822.s006.docx]

**S5 Table. Items used to measure perceived stress in the HRS and the MIDUS samples.**

| **HRS** |
| --- |
| *Perceived Discrimination* |
| You are treated with less courtesy or respect than other people. |
| You receive poorer service than other people at restaurants or stores. |
| People act as if they think you are not smart. |
| People act as if they are afraid of you. |
| You are threatened or harassed. |
| *Perceived Job Stress* |
| My work schedule makes it difficult to fulfill personal responsibilities. |
| Because of my job, I don't have the energy to do things with my family or other important people in my life. |
| Job worries or problems distract me when I am not at work. |
| *Perceived Family Stress* |
| How often does your husband/wife/partner make too many demands on you? |
| How much does your husband/wife/partner criticize you? |
| How much does your husband/wife/partner let you down when you are counting on them? |
| How much does your husband/wife/partner get on your nerves? |
| My home life keeps me from getting work done on time on my job. |
| My family or personal life drains me of the energy I need to do my job. |
| I am preoccupied with personal responsibilities while I am at work. |
| *Perceived Interpersonal Stress* |
| How often do your children make too many demands on you? |
| How much do your children criticize you? |
| How much do your children let you down when you are counting on them? |
| How much do your children get on your nerves? |
| How often does other immediate family make too many demands on you? |
| How much does other immediate family criticize you? |
| How much does other immediate family let you down when you are counting on them? |
| How much does other immediate family get on your nerves? |
| How often do your friends make too many demands on you? |
| How much do your friends criticize you? |
| How much do your friends let you down when you are counting on them? |
| How much do your friends get on your nerves? |
| *Perceived Neighborhood Stress* |
| I really feel part of this area/I feel that I don't belong in this area. |
| There is no problem with vandalism and graffiti in this area/Vandalism and graffiti are a big problem in this area. |
| Most people in this area can be trusted/Most people in this area can't be trusted. |
| People feel safe walking alone in this area after dark/People would be afraid to walk alone in this area after dark. |
| Most people in this area are friendly/Most people in this area are unfriendly |
| This area is kept very clean/This area is always full of rubbish and litter |
| If you were in trouble, there are lots of people in this area who would help you/If you were in trouble, there is nobody in this area who would help you |
| There are no vacant houses or storefronts in this area/There are many vacant or deserted houses or storefronts in this area |
| **MIDUS** |
| How would you rate the amount of control you have over your financial situation these days? |
| During the past year, how often have you thought your relationship might be in trouble? |
| How would you rate your life overall these days? |
| How would you rate the amount of control you have over your life overall these days? |
| Please indicate how strongly you agree that the demands of everyday life often get me down. |
| Please indicate how strongly you agree that what happens in my life is often beyond my control. |
| At present, how much control do you have over your life in general? |
